# Anakinra versus intramuscular methylprednisolone acetate for gout flares in people with chronic kidney disease (ASGARD): a UK randomised multicentre, double-blind, double-dummy controlled feasibility study with economic, qualitative and safety assessment

**DOI:** 10.1136/bmjopen-2025-114454

**Published:** 2026-09-30

**Authors:** Gowrie Balasubramaniam, Mike Almond, Patrick Harnett, David Turner, Jonathan Scales, Jufen Zhang, Carmel Moore, Karen Hayden, Edward Roddy, Christian Mallen, John Stack, Shweta Bhagat, Nora Ng, Andrew Rutherford, Bhaskar Dasgupta

**Affiliations:** 1 Department of Renal Medicine, Mid and South Essex NHS Foundation Trust, Basildon, UK; 2 Anglia Ruskin University, Chelmsford, UK; 3 Department of Renal Medicine, Anglia Ruskin University, Chelmsford, UK; 4 Department of Renal Medicine, Medical Specialist Group LLP, Saint Martin, Guernsey; 5 Department of Renal Medicine, University of East Anglia Norwich Medical School, Norwich, UK; 6 Department of Renal Medicine, University of Essex, Colchester, UK; 7 Research Institute for Primary Care and Health Sciences, Keele University, Keele, UK; 8 Keele University, Keele, UK; 9 Mater Misericordiae University Hospital, Dublin, Ireland; 10 Department of Renal Medicine, West Suffolk Hospitals NHS Trust, Bury Saint Edmunds, UK; 11 Guy’s and St Thomas’ Hospitals NHS Trust, London, UK; 12 Department of Renal Medicine, King’s College London, London, UK; 13 Rheumatology, Anglia Ruskin University, Chelmsford, UK

**Keywords:** Clinical Trial, Adult nephrology, RHEUMATOLOGY

## Abstract

**Objectives:**

Anakinra versus steroids for gout flares in people with renal disease (ASGARD) is a feasibility trial of undertaking a double-blind, double dummy randomised controlled trial of Anakinra versus intramuscular methylprednisolone acetate in people with gout and chronic kidney disease.

**Design:**

A two-arm parallel group, double-blind, double-dummy, multicentre, randomised controlled trial.

**Setting:**

ASGARD was undertaken in four centres in secondary care.

**Intervention:**

Subcutaneous anakinra 100 mg daily for 5 days versus intramuscular methylprednisolone acetate 120 mg for gout flares in people with chronic kidney disease (estimated glomerular filtration rate (eGFR) <60 mL/min/1.73 m^2^).

**Primary and secondary outcome measures:**

Primary outcome measures were feasibility criteria of recruitment (32 participants over 15 months), enrolment of >70% of eligible participants, <10% treatment crossover or failure and completion of 5 out of 7 participant-reported pain assessments by 85% of participants. Secondary outcome measures included time to 50% reduction and complete resolution of pain (self-assessment) on Visual Analogue Scale (0–100 mm) and 5-point Likert scale (D1 to D7), physician assessment of joint tenderness and swelling (D1, D2 and D7), patient-reported outcome measure (D1 to D7), patient assessment of activity limitation and quality of life: Health Assessment Questionnaire Disability Index and 36-Item Short Form Survey (D1, D7 and 8 weeks); EuroQol 5-Dimension 5-Level at D1, D2, D7 and 8 weeks; Lower Extremity Functional Scale at D1, D2, D7. Safety data, health economic data and qualitative analysis of participant experience were also collected.

**Results:**

The feasibility trial did not meet its overall criteria for success due to under-recruitment. It met procedural feasibility of recruiting ≥70% of eligible patients and ≤10% treatment crossovers or failure, 85% of participants completing 5 out of 7 participant-reported pain assessments (excluding four participants who did not receive allocated treatment). 21 participants (mean age 72 years, mean eGFR 44 mL/min/1.73 m^2^) were randomised; 17 received per protocol treatment. Mean reduction in pain after 7 days was 46.8 from baseline by 100 mm visual analogue score. Anakinra appeared to have a shorter time to pain reduction; however, this must be confirmed in a larger trial. Functional and quality of life assessments were highly completed. Health resource use was higher when assessed by health records. Qualitative feedback from participants alluded to high questionnaire burden and delays in receiving treatment.

**Conclusions:**

We demonstrated the feasibility of conducting a definitive randomised controlled trial with insights from treatment effects, safety data, reconciliation of assessments, economic evaluation and participant experience. Improved case finding in the community, with streamlined processes during gout flare should be explored before recommendation for future trial can be made.

**Trial registration numbers:**

EudraCT No. 2015-001787-19. NCT/ClinicalTrials.gov No. NCT02578394. WHO Universal Trials Reference No. U1111-1175-1977.

**STRENGTHS AND LIMITATIONS OF THIS STUDY**

- This study uses a ‘gold-standard’ double-blind, double-dummy study design focusing specifically on gout flares in people with kidney disease.
- This study provides an evaluation for efficacy, safety and broad functional assessments.
- This feasibility study undertakes an economic and qualitative assessment exploring participant experience.
- This study included patients from primary care and secondary care.
- The feasibility study is not powered for efficacy comparison.

## Introduction

 Gout is a common condition that causes acutely painful, debilitating flares that require prompt treatment. Treatment in people with gout and chronic kidney disease (CKD) is challenging due to the risk of drug toxicity and a lack of evidence. A recent literature review^1^ highlighted the lack of efficacy data in people with an estimated glomerular filtration rate (eGFR) <60 mL/min/1.73 m^2^ (eGFR criteria for CKD), and the harm associated with inappropriate prescribing has been described previously.^2 3^

Gout affects 1%–4% of the population in Europe.^4^ The prevalence of CKD in people with gout is about 24%, with patients having a higher risk of flares.^5 6^ Non-steroidal anti-inflammatory drugs have the largest evidence base for treating gout flares but are contraindicated in moderate-to-severe CKD as they can cause acute kidney injury, gastrointestinal bleeding, worsening heart failure, hypertension and liver failure.^2 3^ Colchicine must be used with caution due to multiple drug interactions and a narrow therapeutic index, low-dose colchicine induced diarrhoea in 23%–46% of people with gout without CKD.^7 8^ Glucocorticoids have some evidence for use in people with gout and CKD from older trials.^9^ Triamcinolone is commonly used in the USA and is used as the active comparator in contemporary studies.^9–11^ A double-blind randomised controlled study showed that prednisolone 35 mg for 5 days (total 175 mg) was non-inferior to naproxen for gout flares, although people with moderate to advanced kidney disease were excluded.^12^ The dosing is equivalent to the commonly used treatment in the UK, Depo-Medrone at 120 mg (175 mg prednisolone is equivalent to 140 mg methylprednisolone acetate).^13^

Interleukin-1 signalling plays a key role in gout-associated inflammation^14^ and agents targeting this pathway have shown efficacy in treating gout flares.^10 11 15–23^ Anakinra is a recombinant human interleukin-1 receptor antagonist. A pilot study of its use in gout flares showed prompt resolution of symptoms and included three people with serum creatinine readings of 121, 204 and 406 mmol/L.^21^ Two randomised controlled studies have confirmed efficacy, one study compared Anakinra 100 mg once daily for 5 days against standard treatment (colchicine 500 mcg twice a day or naproxen 500 mg twice a day or prednisolone 35 mg once a day for 5 days).^23^ The Anago study,^16^ compared two doses of Anakinra, 100 mg once a day or 200 mg once a day for 5 days against triamcinolone 40 mg as a single injection, and showed that Anakinra 100 mg once a day had comparable efficacy. Both studies excluded people with severe kidney disease as defined by an eGFR <30 mL/min/1.73 m^2^.

The Anakinra versus steroids for gout flares in people with renal disease (ASGARD) feasibility trial is a randomised multicentre, double-blind, double-dummy controlled trial of Anakinra 100 mg subcutaneous for 5 days compared with a one-off methylprednisolone acetate 120 mg intramuscular injection. The primary aim is to assess the feasibility of undertaking a definitive trial in people with gout flare and CKD. The objectives were to test recruitment and retention rates; willingness of potential participants to be randomised; test eligibility criteria; collect and analyse outcome data to inform sample and power calculations for a trial of efficacy; collect economic data to inform a future economic evaluation estimating costs of treatment and assess capacity of the project to scale up to a larger national multicentre trial. We also aimed to gather insight from participants about the trial involvement in a nested qualitative study.

## Methods

The methods for this feasibility trial have previously been published.^24^ Briefly, we aimed to recruit participants from six centres in the East and Southeast of England. Participants were recruited from secondary care by referral from primary care or were already in hospital and developed a gout flare. People known to have CKD and gout were sent information about the feasibility trial, primary care sites were used as participant identification centres to help recruit potential participants before an acute flare by sending invitation letters. Gout flare was confirmed by American College of Rheumatology 1977 criteria for acute arthritis of gout. Participants were randomised in a 1:1 ratio using blocks of four with an internet-based randomisation system. Allocation concealment and blinding were maintained throughout the trial, including data analysis. Anakinra and its placebo were supplied by the manufacturer. Lipofundin was used as the placebo for intramuscular methylprednisolone acetate,^11^ with the syringes for injection prepared by a member of the trial not involved with randomisation.

One treatment arm received Anakinra 100 mg by subcutaneous injection for 5 days and a single placebo methylprednisolone acetate (lipofundin) intramuscular (gluteal) injection on day 1. The other treatment arm received a single intramuscular methylprednisolone acetate (Depo-Medrone) 120 mg and placebo Anakinra by subcutaneous injection for 5 days.

The dose of Anakinra 100 mg was chosen based on published literature at the time and has been reaffirmed by subsequent studies. Depo-Medrone was used as control as it is the most used treatment in the UK for gout flares (surveyed in our region).^25^ A dose of 120 mg was used due to its equivalence of oral dosing prednisolone 35 mg for 5 days. Oral prednisolone 35 mg orally for 5 days was available as rescue treatment in both groups.

This feasibility trial was not powered for inference. We undertook exploratory testing of primary and secondary end points to look for differences in the anakinra group versus the methylprednisolone acetate group. Primary end points explored were time to 50% reduction and complete resolution of pain in self-assessed pain intensity in the joint most affected measured on the Visual Analogue Scale^26^ (VAS) (0–100 mm). A 5-point Likert scale^26^ for pain (none, mild pain, moderate pain, severe pain, extreme pain) from baseline (day 1) to day 7 post randomisation was also explored. The secondary end points explored were physician assessment of joint tenderness and swelling (days 1, 2 and 7) using a 4-point Likert scale.

The sample size was informed by previously published literature and^27^ statistical analysis was prespecified in the protocol (supplement, protocol section 10.3). The feasibility criteria for success were recruit ≥70% of eligible people and meet recruitment target (15-month period); 85% of participants completing 5 out of 7 participant-reported pain assessments and ≤10% treatment crossovers or failure.

### Functional assessment and health-related quality of life

Assessment of activity limitation and quality of life were undertaken using the Health Assessment Questionnaire Disability Index (HAQ-DI)^28^ and 36-Item Short Form Survey (SF-36) at days 1, 7 and 8 weeks^29^; five-level EuroQol questionnaire^30 31^ (EuroQol 5-Dimension 5-Level, EQ-5D-5L) at days 1, 2, 7 and 8 weeks and Lower Extremity Functional Scale (LEFS)^32^ at days 1, 2 and 7. The rationale for the use of possible overlapping assessments was to enable reconciling assessments for a subsequent definitive study based on completion and responsiveness to treatment effect.

### Health economics

We tested methods and processes for collecting health economic data, and recorded resources required for drug treatment in both groups including costs of drugs and administration. We also recorded healthcare contacts by participant administered questionnaire at the 8-week follow-up. Where possible, we also recorded the use of healthcare from the participant’s records from secondary care and other sources to compare these two different methods of collecting resource use data. Costs of inpatient stays were estimated using a weighted average of all non-elective inpatient stays, taken from NHS reference costs.^33^ Costs for Accident and Emergency and outpatient visits were taken from the same source using weighted averages. For outpatient costs, we had sufficient data to assign a specialty to these costs, these were assigned a weighted average for the appropriate specialty. Costs for primary care contacts (general practitioner, practice nurses, district nurses) were taken from costs published by the University of Kent.^34^ Drug costs were taken from the BNF website.^35^ All costs are in 2019/2020 UK pounds Stirling. As the trial had a follow-up of 8 weeks neither cost was outcomes discounted. The cost of the intervention included the costs of the drugs and some allowance for staff time. The first injection and some of the subsequent injections were given by a nurse, the mean number of injections given by a clinician in the Anakinra group was 1.75. We assumed 15 min of (grade 5) nurse time and only included costs that would have been required to provide the randomised intervention.

The outcome measure used in the economic evaluation was the EQ-5D-5L.^30 31^ This was scored using the ‘cross walk’ value set,^31^ as recommended by the National Institute of Health and Clinical Excellence at the time of analysis.^36^

### Biochemical markers

Measure of blood markers of inflammation and kidney function including urea and electrolytes, C reactive protein and white blood cell counts were undertaken at baseline (day 1), day 2 and day 7. We also measured urate and liver function tests.

### Qualitative analysis

All participants who gave their specific informed consent for participation in a qualitative study were selected for an interview. Qualitative feedback from twelve participants was planned by telephone interview (participants interview questions in online supplemental file 2 pg. 15) with a trained qualitative researcher to gain experience of taking part in the feasibility trial. Interviews were transcribed and analysed thematically and aspects that may strengthen future trial design.

### Patient and public involvement

Patient and public involvement (PPI) was undertaken as part of the grant application for key aspects of the study design including the burden of the intervention and assessments. PPI involvement was throughout the study with insight gained from ways of improving recruitment. Feedback will be given on research outputs from the study.

## Results

### Participants

The first participant was recruited on 14 December 2016 and further ethics approved protocol changes were required to the inclusion protocol for clarity and introduction of prescreening to expedite assessment. 16 participants were recruited between 5 May 2017 and 5 June 2019, there was then a halt to recruitment and further ethics approved modifications to the protocol following the COVID-19 pandemic to minimise risk to participants. Four participants were recruited from 5 June 2019 to the end date of 4 January 2022. Key amendments to the protocol are listed in the table in appendix. 20 out of the 21 people with gout were randomised from the primary site, with many sites having had to be closed due to challenges in recruiting participants.

There were 310 potential participants prescreened/screened from the four different sites (two sites did not collect prescreening data) (figure 1). Of the 289 that were not recruited, the reasons for non-eligibility were recorded in 203 potential participants. The most common cause recorded was lack of eGFR <60 mL/min/1.73 m^2^ (101/203), the next most common causes were no gout diagnosis (19/203), no gout flare (17/203), and on colchicine (17/203).

**Figure 1:**
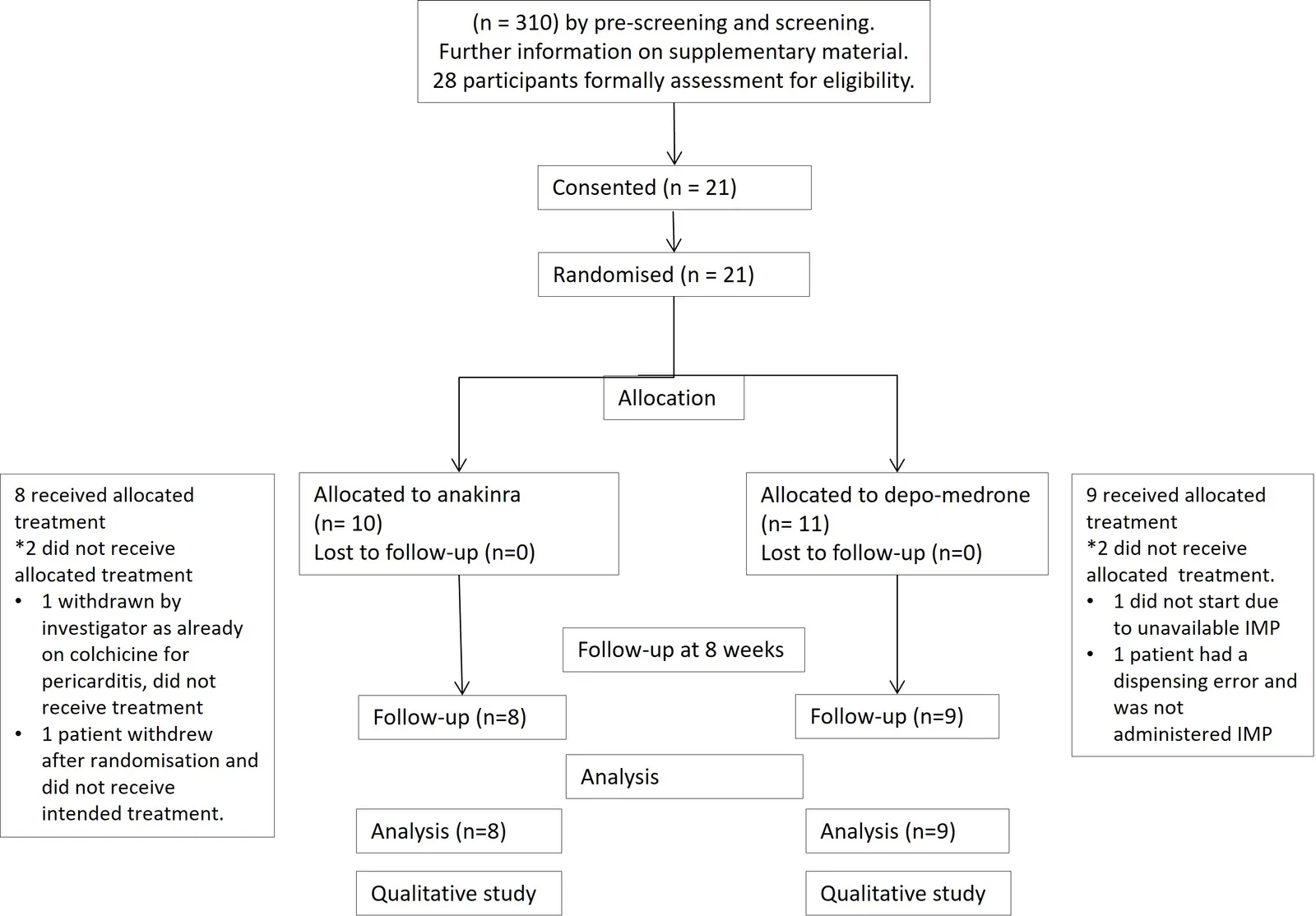
Participant flow diagram in the ASGARD feasibility trial. *Four participants did not receive protocol treatment and were excluded from efficacy analysis. ASGARD, Anakinra versus steroids for gout flares in people with renal disease; CONSORT, Consolidated Standards of Reporting Trials; IMP, investigational medicinal product.

21 participants were enrolled and randomised (figure 1). 10 were allocated to the Anakinra arm and 11 were allocated to the Depo-Medrone arm. Four were recruited from in-patient presentation (Anakinra 1, Depo-Medrone 3). One participant in the Anakinra arm had to be withdrawn as they received colchicine for another condition, and one withdrew prior to receiving investigational medicinal product (IMP) as they were concerned about interaction with diabetes. Eight out of the 10 participants in the anakinra arm completed assessment to week 8. Two participants in the Depo-Medrone arm had to be withdrawn due to pharmacy issues, one due to unavailability of the IMP and the other did not receive IMP due to a dispensing error. Nine out of the 11 participants in the Depo-Medrone arm completed assessment to week 8.

Participant baseline characteristics are listed in table 1, just over half were male and the mean age was 71.9 years with an SD of 12.0. The mean eGFR was 43.5 mL/min/1.73m^2^; two participants in the anakinra arm had an eGFR <30 mL/min/m^2^. Only two participants had been on urate lowering therapy even though 16 had a history of recurrent flares. The first metatarsal interphalangeal joint was affected in 10 participants, with three others involving the ankle and midfoot. Only one participant underwent synovial fluid analysis. The four participants who did not receive allocated treatment were excluded from analyses.

**Table 1:** Table showing baseline demographics and characteristics of participants who received treatment

	Anakinra	Depo-Medrone
	(n=8)	(n=9)
Age (years), mean (SD)	69.3 (15.1)	70.6 (10.1)
Male, n	5	3
Inpatient	1	3
Outpatient	7	6
Race		
White	7	8
Black	0	1
Asian	1	0
Comorbidities		
Type 2 diabetes	3	4
IHD	0	1
Heart failure	2	3
Hypertension	5	8
Smoking history	3	5
Baseline		
Creatinine: mean (SD)	170.8 (110.6)	130.2 (34.3)
eGFR mL/min/1.73 m^2^	41.8 (17.2)	41.8 (9.5)
30–60	6 (75%)	9 (100%)
<30	2 (25%)	0 (0%)
CRP: mean (SD)	15.9 (17.8)	30.2 (31.0)
Urate: mean (SD)	534.4 (102.0)	547 (135.7)
Urate lowering therapy	2	0
MSU crystals in symptomatic joint or tophus	0	1
Joint involved		
MTP	2	7
Ankle or midfoot	1	1
Other than above	5	1
First episode	0	2
Recurrent episode	8	6
Clinical evidence of tophi	2	2
Synovial fluid analysis of a symptomatic (ever) joint or bursa (MSU negative)	0	1
Imaging evidence of urate deposition in symptomatic (ever) joint or bursa (present)	0	0

MTP, metatarsophalangeal.CRP, C reactive protein; eGFR, estimated glomerular filtration rate; IHD, ischaemic heart disease; MSU, monosodium urate.

### Primary outcomes

The feasibility trial did not meet its criteria for success due to not meeting recruitment target of recruiting 32 patients over 15 months. Other elements of procedural feasibility (recruit eligible participants, data completion, treatment crossover and failure) were met. We recruited ≥70% of eligible participants with ≤10% treatment crossovers or failure and more than 85% of participants randomised completed five out of seven participant-reported pain assessments. The latter would not have been met if we include the four participants who did not receive allocated treatment due to protocol and pharmacy issues.

#### Proposed primary outcome: measures of pain

The 0–100 VAS performed well demonstrating improvement in pain in both groups with a fall in VAS score from baseline (day 1) to days 2 to 7 as shown in table 2. The mean change from baseline to day 3, day 5 and day 7 was 29.1 vs 15.2, 40.8 vs 26.9, and 46.3 vs 47.2 in the Anakinra arm versus the Depo-Medrone arm, respectively. The Kaplan-Meier curve for 50% reduction in VAS from baseline with a proposed power calculation is provided in the online supplemental file 2.

**Table 2:** Table showing mean change in 0–100 mm VAS from baseline

	Anakinra		Depo-Medrone	
	Mean (SD) (n=8)	Change from baseline mean (95% CI)	Mean (SD) (n=9)	Change from baseline mean (95% CI)
Day 1	67.25 (9.5)	–	69.3 (11.2)	–
Day 2	44.1 (16.1)	−23.1 (7.3 to 39.0)	58.4 (12.3)	−10.9 (−1.7 to 23.5)
Day 3	38.1 (17.9)	−29.1 (11.9 to 46.3)	54.1 (21.1)	−5.2 (−4.4 to 34.8)
Day 4	33 (14.0)	−34.3 (20.3 to 48.2)	43.7 (23.5)	−25.7 (4.9 to 46.4)
Day 5	26.5 (16.2)	−40.8 (24.2 to 57.3)	42.4 (23.9)	−26.9 (6.0 to 47.8)
Day 6	24.5 (13.6)	−42.8 (28.6 to 56.9)	35.7 (25.7)	−33.7 (10.9 to 56.4)
Day 7	21 (13.2)	−46.3 (31.3 to 61.2)	22.1 (16.1)	−47.2 (31.6 to 62.9)

VAS, Visual Analogue Scale.

The participant assessment of pain and physician assessment showed improvement overall with no differences between treatment arms. Participant assessment of pain improved from baseline to day 7. The Likert scores showed a mean change of −1 at day 2 and −2 at day 7. Physician assessment of joint swelling and tenderness showed a similar pattern (online supplemental file 2).

### Functional assessment and health-related quality of life

The LEFS showed improvement from day 1 to day 7 (table 3). The HAQ-DI showed some improvement from day 1 to day 7, with a subsequent decline (table 3). Assessment of discrimination between treatment groups could not be obtained due to low numbers (supplement).

**Table 3:** Table showing overall trends in functional assessment and quality of life

	VAS	LEFS	HAQ-DI	EQ-5D-5L	SF36 (PCS)	SF36 (MCS)
Day 1 (baseline)	70 (61–73)	22.5 (14.5–35.5)	1.3 (0.6–1.5)	0.3 (0.04–0.7)	22.1 (21.8–28.9)	42.2 (37.7–57.4)
Day 2	56 (40–64)	25 (14-37)	–	0.5 (0.2–0.6)	–	–
Day 7	23 (11–28)	33 (20-50)	1 (0.9–1.4)	0.7 (0.6–0.72)	29 (25.3–31.5)	40.7 (36.2–56.7)
Week 8	–	–	1.3 (0.3–2.1)	0.6 (0.2–0.7)	32.3 (24.4–44.9)	49.8 (34.8–55.3)

Median (IQR).

MCS, Mental Component Summary.EQ-5D-5L, EuroQol 5-Dimension 5-Level; HAQ-DI, Health Assessment Questionnaire Disability Index; LEFS, Lower Extremity Functional Scale; SF-36, 36-Item Short Form Survey; SF-36, 36-Item Short Form Survey; VAS, Visual Analogue Scale.

All participants who completed protocol treatment completed quality of life measures. Differences between groups were difficult to assess but the overall results showed a low mean baseline for the SF-36 and the HAQ-DI. The EQ-5D-5L score showed a trend of improvement from baseline to day 7 with a slight decline in week 8, like the HAQ-DI. The trends of the LEFS, HAQ-DI and the physical component of the SF-36 were comparable up to day 7 (supplement). The overall SF-36 physical component performed best with regards to a trend for improvement mirroring the VAS (table 3).

#### Biochemical markers

Blood parameters of inflammation showed lower levels of C reactive protein (CRP) and neutrophils in the Anakinra group, especially at day 7 (mean±SD: neutrophils 3.3±1.06 vs 6.3±1.95; CRP 3.9±1.84 vs 20.7±20.02). Haemoglobin, platelet count, urate and liver function tests did not change (online supplemental file 2).

#### Rescue medications and adverse events

No participants received rescue medications during 7-day assessment phase. Six participants had further flares, four in the Anakinra arm and two in the Depo-Medrone arm. There were no serious adverse events. There were 19 adverse events reported in total, 14 in the Anakinra arm and 5 in the Depo-Medrone arm. One participant in the anakinra arm reported seven adverse events. Details of gout flares and adverse events are given in online supplemental material.

#### Health economics

Total estimated intervention costs were £141 (£129 to £153) and £16.50 (cost same for all individuals) for Anakinra and Depo-Medrone, respectively. These costs assumed that packs of seven vials for anakinra could be split between participants. If a single pack of seven vials was needed for each participant with the remaining two vials discarded, then the estimated cost for Anakinra would increase to £161 (£152 to £171).

Healthcare usage after 8 weeks is given in table 4. There is some overlap between hospital records data and self-report data as some events were reported in both sources. Results are not presented by group because of the large degree of heterogeneity in the results. In-patient stay was a key driver of cost and was better recorded using electronic health records.

**Table 4:** Table showing healthcare usage after 8 weeks

	Records			Self-report		
Service use	Events	Days	Cost	Events	Days	Cost
Initial inpatient stay	3	56	£10 557			
Follow-up stays	6	86	£21 114	5	67	£17 595
Outpatient	5		£746	3		£408
A&E	1		£155	1		£155
Day hospital				2		£1626
GP				11		£432
District nurse				34		£1009
Other				2		
Home help				34		£816
Total secondary care			£32 572			£19 784
Total primary care						£3882

A&E, Accident and Emergency; GP, general practitioner.

### Qualitative study

Seven trial participants (ages ranging from 56 to 81) were interviewed by telephone to gain a qualitative understanding of their experience of participating in the feasibility trial. This was reduced from a planned twelve interviewees due to poor recruitment into the trial. Four women and three men living in various types of domestic situation (with/without spouse/car) were interviewed. One interview was conducted with the participant’s husband as the participant was herself too tired to undertake a telephone call.

Interviewees were all very positive about their experience of taking part in the trial. They felt that the participant information (online supplemental file 2) they were given was clear and comprehensive, and emphasised the care and time that the recruiting clinicians put into answering their questions. Most interview respondents reported that they understood the participant information materials including the meaning of randomisation and placebo, one participant was uncertain about the double dummy nature of the trial. There were reports of delay from recruitment to receiving their first injection on day one (up to 7 hours). Participants generally self-injected at home with no problem and felt their preparation for this by clinicians gave them the confidence needed to do so without worry. Around half of participants found keeping the daily diary burdensome and frustrating. Further information on qualitative study is available as online supplemental file 2.

## Discussion

This feasibility trial gives insights into designing trials for people with gout flare and CKD who tend to be older with comorbidities. This is relevant due to an ageing population and the projected health and economic burden.^37 38^ We experienced numerous recruitment barriers, even with the increasing burden of these conditions,

The first barrier was in participant identification in primary care, our screening data suggested that about half of the prescreened/screened people with gout were excluded due to not having a sufficiently low eGFR to have a diagnosis of CKD. CKD identification and registering have been known to be poorly undertaken in primary care^39^ but this information would be difficult to confirm to see if it has an impact in this study. There is now an increasing impetus to identify patients with CKD due to numerous treatments that have been shown to reduce CKD progression. The second barrier to recruitment was the trial logistics of undertaking a study during an acute presentation like gout. The IMP can only be dispensed from secondary care, so all participants had to be seen in a secondary care setting. Presentations to acute services may occur via multiple pathways in complex clinical contexts, especially in older people, and it may be difficult to solely focus on the gout flare. Recruitment did not markedly improve even after the eligibility criteria were broadened to include recruitment on the following day after admission (amendments to protocol, supplement). The limited availability of the trial team to working hours and the need for two research nurses to maintain blinding, with a medical doctor for consenting, and trial pharmacist to dispense the drug imposes logistical challenges for trial procedures. These would contribute to delays in treating an acute pain flare which would be the priority of the clinical treating team. This is reflected in the lower recruitment from in-patients even though there is an increasing number of presentations to secondary care.^40^ Participants who were referred from primary care would have received a more streamlined experience with appropriate preparation of relevant trial team but still experienced marked delays in receiving medications as suggested by qualitative survey (online supplemental file 1). Ways to potentially overcome these barriers with future study design may be to undertake a more pragmatic approach. Modifiable aspects of the trial would include elements prior to drug dispensing. Case finding from primary care could be undertaken in a manner used in the CONTACT trial;^8^ eligible patients who have had gout flares are contacted regularly and consented remotely in anticipation of a flare, streamlining as much trial activity prior to attending secondary care for assessment and dispensing of trial drug. These changes would have to be considered in a subsequent feasibility assessment and would preclude progression of this design to a definitive trial.

Most recruitment occurred at the chief investigator’s site which adds further limitation to the trial; many sites were closed due to lack of recruitment. Aspects mentioned above with challenges to recruitment may have contributed to lack of recruitment at these sites. This was further compounded by drug delivery issues and the COVID-19 pandemic. The trial end date was also extended due to the COVID-19 pandemic and increasing difficulty in getting a further bulk delivery of IMPs due to Britain leaving the European Union.

The mean age in our trial is significantly higher than any other acute gout study and this may have impacted on the baseline clinical metrics with higher HAQ-QI scores and lower SF-36 scores compared with previous published data. Use of urate lowering therapy in participants with recurrent flare was poor, potentially due to concerns about toxicity in people with kidney disease.

Anakinra appeared to improve VAS score earlier than Depo-Medrone, a power calculation for a trial of superiority that may confirm this once recruitment barriers have been overcome has been suggested in the supplement. The Anago study^16^ did not show any difference in the primary endpoint of changes in VAS to 24–72 hours but did suggest differences when looking at time to onset of pain reduction. This is like our trial with Anakinra against Depo-Medrone, showing a >20% reduction at day 2 vs day 3 from baseline (day 1), and >50% reduction at day 4 vs day 6 from baseline (day 1), respectively. Our trial shares some similarity with the canakinumab trial which also had a high number of patients with comorbidities and CKD, and a difference in the reduction of VAS at 72 hours was demonstrated.^11^ This may be due to a lower ceiling effect in the VAS in this patient group.

The OMERACT group endorses the generic SF-36 and HAQ-DI for acute gout studies, but these have considerable floor and ceiling effects,^26^ we would anticipate that a group with a worse baseline function would further add to such an effect. Our data suggests that the HAD-QI ceiling effect and the SF-36 and LEFS floor effect may make them less responsive. Our feasibility trial suggests that the LEFS may not offer better discrimination than the EQ-5D-5L and SF-36. The EQ-5D-5L was highly completed and returned; the day 2 score may not offer additional discrimination over the SF-36. The resolution to a poorer score after treatment may reflect the poor baseline function and quality of life in this group of patients due to comorbidity and hospitalisation.

The qualitative study pointed to delays in treatment and assessment burden. Considering all factors, we would recommend using VAS for primary outcome assessment of pain from day 1 to day 7, with a time to >50% reduction in VAS as the primary outcome measure, and the EQ-5D-5L at days 1, 7 and 8 weeks for functional and quality of life assessments.

Anakinra reduced levels of CRP, like the Anago study,^16^ and neutrophils at day 7. We did not observe a fall in white blood cells, neutrophils, platelets, and urate that occurred in with canakinumab^15^ but there was a difference seen in the levels of neutrophils and white cells between our treatment arms due to a small decrease in the Anakinra arm and a rise in the Depo-Medrone arm.

There was an imbalance in safety events between groups as one individual reported multiple adverse events. The nature of the double blind, double dummy design means that this cannot be fully dismissed, however, the non-specific nature of the symptoms and the low participant number make it difficult to interpret conclusively. We feel that this imbalance in events should not preclude a larger trial but could be mentioned in an investigator brochure for awareness.

Our trial showed much higher values recorded for health resource use from health records compared with self-report which was primarily due to the in-patient costs. Self-reported data asked for inpatient stays in the last 8 weeks so it is not clear if any initial inpatient stay would be covered but it did capture community health resource use. A future study should obtain data on secondary care usage from notes where possible. Anakinra is more expensive than steroids but overall cost for health use in this group of patients is high, especially secondary care costs. It would be important for the definitive trial to include an economic evaluation so that any additional costs could be considered alongside potential savings from reductions in length of stay and any health benefits that may arise from Anakinra.

This is the first trial to compare treatments for gout flares specifically in people with kidney disease (eGFR <60 mL/min/1.73 m^2^). It also recruited from an in-patient population, capturing a vulnerable cohort of people who experience gout flares, and it is the first use of Anakinra and steroids in participants with advanced kidney disease with eGFR <30 mL/min/1.73 m^2^ in a trial setting with safety monitoring. To our knowledge, this is the oldest trial population in a gout flare trial reported in the literature.

## Conclusions

The ASGARD study feasibility criteria were not met due to failure to recruit to target but procedural feasibility aspects were met, namely, to recruit 70% of eligible participants, 85% of participants who received treatment completed assessments and there was no more than 10% treatment failure or cross-over. The VAS performed better than the Likert score with regards to discrimination between outcomes and should be used as the primary outcome. The time to 50% reduction may represent the appropriate primary outcome measure for a future definitive study. The study suggested an achievable sample size with good power for a subsequent trial of superiority if recruitment barriers can be overcome, these interventions would require new feasibility assessment. We suggest using the EQ-5D-5L at days 1, 5 and week 8 with economic assessment using health records. Interview respondents were generally very positive about the way the trial was run and the care they received, although they expressed some points on delays and questionnaire burden. Consideration should therefore be given in any future trial to streamline processes and rationalise questionnaires.

## Supplementary material

10.1136/bmjopen-2025-114454 online supplemental file 1

10.1136/bmjopen-2025-114454 online supplemental file 2

## Data Availability

Data are available on reasonable request.
