# Supplementary material for: Anakinra versus intramuscular methylprednisolone acetate for gout flares in people with chronic kidney disease (ASGARD): a UK randomised multicentre, double-blind, double-dummy controlled feasibility study with economic, qualitative and safety assessment
Source: BMJ Open. 2026 Sep 30;16(9):e114454. doi: 10.1136/bmjopen-2025-114454 (PMC13629947; doi:10.1136/bmjopen-2025-114454)
Supplement: Supplementary file 2 [file bmjopen-2025-114454.reviewer_comments.pdf]

PEER REVIEW HISTORY

BMJ Open publishes all reviews undertaken for accepted manuscripts. Reviewers are asked to complete a checklist review form and are provided with free text boxes to elaborate on their assessment. These free text comments are reproduced below.

ARTICLE DETAILS

Title (Provisional)

Anakinra versus intramuscular methylprednisolone acetate for acute gout flares in people with chronic kidney disease (ASGARD): a United Kingdom randomised multicentre, double-blind, double-dummy controlled feasibility study with economic, qualitative and safety assessment.

Authors

Balasubramaniam, Gowrie; Almond, Mike; Harnett, Patrick; Turner, David; Scales, Jonathan; Zhang, Jufen; Moore, Carmel; HAYDEN, Karen; Roddy, Edward; Mallen, Christian; Stack, John; Bhagat, Shweta; Ng, Nora; Rutherford, Andrew; Dasgupta, Bhaskar

---

VERSION 1 - REVIEW

**Reviewer**	**1**
**Name**	**Kongwatcharapong,, Junporn**
**Affiliation**	**Mahidol University**
**Date**	**19-Jan-2026**
**COI**	**None**

This manuscript reports a multicentre, double-blind, double-dummy feasibility randomised controlled trial comparing anakinra with intramuscular methylprednisolone acetate for acute gout flares in individuals with chronic kidney disease (CKD). The topic addresses a clear evidence gap, as patients with moderate-to-severe CKD are frequently excluded from gout flare trials despite high clinical need.

The study is appropriately framed as a feasibility trial, and the authors are generally careful not to overstate efficacy findings. The inclusion of qualitative and health economic components is a particular strength and aligns well with BMJ Open's scope. However, several areas require clarification or strengthening to improve transparency, internal consistency, and usefulness for informing a future definitive trial.

Overall, this is a methodologically sound feasibility study, but revisions are required before the manuscript is suitable for publication.

Major comments

1. Framing of feasibility objectives and success criteria

The feasibility objectives are clearly listed, and predefined success criteria are described. However:

-The manuscript reports that three out of four feasibility criteria were met, but the interpretation of partial success would benefit from more explicit reflection.

-The recruitment shortfall (21 recruited vs. target of 32 over 15 months) is substantial and should be more clearly framed as a key feasibility limitation, rather than balanced by other successes.

Suggestion:

Explicitly distinguish between procedural feasibility (data completion, blinding, crossover rates) and recruitment feasibility, and clarify whether the latter alone would preclude progression to a definitive trial without major design changes.

2. Recruitment challenges and external validity

Recruitment difficulties dominate the results and discussion, yet several issues remain insufficiently explored:

-Most participants were recruited from a single centre, limiting generalisability.

-Many sites were closed due to low recruitment, but site-level barriers are not systematically analysed.

-The manuscript suggests under-recording of CKD and gout in primary care, but this remains speculative.

Suggestion:

Consider presenting a short structured summary of recruitment barriers (e.g. clinical pathway delays, staffing, flare timing, IMP logistics) and explicitly state which barriers are modifiable in a future trial.

3. Interpretation of exploratory efficacy signals

Although the authors repeatedly note that the study was not powered for efficacy, some language implies comparative benefit:

-Statements such as “anakinra appeared to perform better based on VAS scoring” risk being over-interpreted.

-Kaplan–Meier curves and time-to-event analyses are presented, yet the implications for clinical decision-making remain unclear.

Suggestion:

Tighten language throughout Results and Discussion to consistently frame these findings as hypothesis-generating only, and avoid comparative phrasing that could be misread as evidence of superiority.

4. Outcome measures and assessment burden

The evaluation of multiple pain, function, and quality-of-life instruments is valuable. However:

-The rationale for selecting and retaining multiple overlapping instruments (VAS, Likert, SF-36, HAQ-DI, LEFS, EQ-5D-5L) could be clearer.

-Qualitative findings indicate substantial participant burden, which contrasts with the breadth of quantitative assessments used.

Suggestion:

Provide a clearer recommendation for a reduced core outcome set for a future trial, explicitly justified by completion rates, responsiveness, and participant feedback.

5. Safety reporting in a high-risk population

Safety data are reported descriptively, but interpretation could be strengthened:

-The imbalance in adverse events between groups (14 vs. 5) is noted but not discussed in depth.

-Inclusion of participants with eGFR <30 mL/min/1.73m^2 is important, but safety conclusions in this subgroup should be cautious.

Suggestion:

Clarify whether adverse events were clustered within individuals or related to specific mechanisms, and avoid broad safety reassurance given the small sample size.

Minor comments:

1. Abstract and key messages

-The statement “Anakinra is a safe option for people with advanced kidney disease” is too strong for a feasibility study and should be softened.

2. Methods clarity

-Clarify whether exploratory statistical comparisons were prespecified in the protocol or conducted post hoc.

3. Tables and supplementary material

-Some tables (e.g. functional outcomes) could be moved entirely to the supplement to improve readability.

4. Terminology

-Ensure consistent use of “feasibility trial” rather than “trial” alone when interpreting results.

5. Language and style

-Minor grammatical and typographical errors are present and should be corrected during revision. Such as;

Abstract:

Result section

- “≤10% of participant’s treatment crossovers or failure”

Suggested correction: “≤10% of participants experiencing treatment crossover or failure”

-“excluding 4 did not receive allocated treatment”

Suggested correction: “excluding four participants who did not receive allocated treatment”

Conclusion section

-“conducting a definitive randomised controlled”

Suggested correction: “conducting a definitive randomised controlled trial”

Introduction:

-“NSAIDS” suggested correction: “NSAIDs”

-“a multiple drug interactions” suggested correction: “multiple drug interactions”

Methods:

-“randomised 1:1 in blocks of four” suggested correction: “randomised in a 1:1 ratio using blocks of four”

-“The sample size was obtained from previous published literature” suggested correction: “The sample size was informed by previously published literature”

Results:

-“Sixteen participants between 5/5/17 and 05/06/19” suggested correction: “Sixteen participants were recruited between 5/5/17 and 05/06/19”

-“The four participants who did not receive allocated treatment and were excluded from analysis.” suggested correction: “The four participants who did not receive allocated treatment were excluded from the analysis.”

Functional assessment and quality of life:

-“The LEF score showed improved from day 1 to day 7” suggested correction: “The LEFS score showed improvement from day 1 to day 7”

-“The HAQ-DI showed some improved from Day 1 to day 7” suggested correction: “The HAQ-DI showed some improvement from day 1 to day 7”

Discussion:

-“Our trial shares some similarity the Canakinumab trial” suggested correction: “Our trial shares some similarity with the canakinumab trial”

-“Our feasibility trial suggests that LEFS may not offer better discrimination” suggested correction: “Our feasibility trial suggests that the LEFS may not offer better discrimination”

-“performed better that the Likert score” suggested correction: “performed better than the Likert score”

General style and consistency: Inconsistent use of:

-“depo-medrone” vs “Depo-Medrone”

-“day 1” vs “Day 1”

-“eGFR<60” vs “eGFR < 60”

-Minor spacing issues around units (e.g. “mls/min/1.73m2” vs “mL/min/1.73 m^2”)

Suggested correction:

Apply consistent formatting according to BMJ Open style across the manuscript.

**Reviewer**	**2**
**Name**	**M Gonzalez, Fernando**
**Affiliation**	**University of Chile, Nephrology**
**Date**	**28-Apr-2026**
**COI**	**None**

Comments

The project is welcome for the reasons explicitly stated by the authors. There is a gap in knowledge regarding the optimal therapy—considering both efficacy and safety—for gout flares in patients with chronic kidney disease (CKD), particularly those with a glomerular filtration rate below 30 mL/min.

The objective of the project is clear: to explore the feasibility of conducting a sufficiently large study to determine the best treatment for gouty arthritis in patients with CKD, including economic data.

The methodology is very well structured. It is well justified, organized, and clearly describes the intended aim, namely to explore the feasibility of a larger-scale study. The economic component is also very comprehensive.

The results are well described. Unfortunately, the actual number of patients recruited was very small and, as the authors themselves noted in the methodology, it is not possible to

draw conclusions regarding comparative efficacy, although no patient required rescue therapy with oral steroids.

The preliminary economic analysis shows a marked asymmetry in costs between the two therapies used. At first glance, anakinra is considerably more expensive; therefore, it is necessary to identify potential competitive advantages of this biologic agent over injectable steroids that could support a more favorable cost-effectiveness analysis than that suggested at first glance.

The discussion, references, figure, and tables are adequate.

In summary, this is a very interesting project. Achieving sufficient patient recruitment will be challenging, but it is worth attempting.

VERSION 1 - AUTHOR RESPONSE

Response to reviewers

Reviewer 1

1) The framing is corrected to show that it did not meet criteria for success and state firstly that it did not meet criteria for success but adopted suggested wording of procedural success

Abstract change

Results: The feasibility trial did not meet its overall criteria for success due to under recruitment. It met procedural feasibility of recruiting ≥ 70% of eligible patients and ≤ 10% of participants treatment crossovers or failure, 85% of participants completing 5 out of 7 participant-reported pain assessments (excluding four participants who did not receive allocated treatment).

Main result section

The feasibility trial did not meet its criteria for success due to not meeting recruitment target of recruiting 32 patients over 15 months. Other elements of procedural feasibility (recruit eligible participants, data completion, treatment crossover and failure) were met. We recruited ≥ 70% of eligible participants with ≤ 10% of participant's treatment crossovers or failure and more than 85 % of participants randomised completed 5 out of 7 participant-reported pain assessments.

2) Recruitment challenges and external validity

CKD identification in primary care has been referenced with a publication as opposed to the NHS data website to give account of low rates of CKD identification in primary care.

The first barrier was in participant identification in primary care, our screening data suggested that about half of the pre-screened/screened people with gout were excluded due to not having a sufficiently low eGFR to have a diagnosis of CKD. CKD identification and registering have been known to be poorly undertaken in primary care39 but this information would be difficult to confirm to see if it has an impact in this study. There is now an increasing impetus to identify patients with CKD due to numerous treatments that have been shown to reduce CKD progression. The second barrier to recruitment were the trial logistics of undertaking a study during an acute presentation like gout. The investigational medicinal product can only be dispensed from secondary care, so all participants had to be seen in a secondary care setting. Presentations to acute services may occur via multiple pathways in complex clinical contexts, especially in older people, and it may be difficult to solely focus on the gout flare. Recruitment did not markedly improve even after the eligibility criteria were broadened to include recruitment on the following day after admission (amendments to protocol, supplement). The limited availability of the trial team to working hours and the need for two research nurses to maintain blinding, with a medical doctor for consenting, and trial pharmacist to dispense the drug imposes logistical challenges for trial procedures. These would contribute to delays in treating an acute pain flare which would be the priority of the clinical treating team. This is reflected in the lower recruitment from in-patients even though there is an increasing number of presentations to secondary care40. Participants who were referred from primary care would have received a more streamlined experience with appropriate preparation of relevant trial team but still experienced marked delays in receiving medications as suggested by qualitative survey (supplemental material).

3) A section is added on possible barriers and how they may be overcome, we have suggested that all these changes would preclude the future success of a larger trial in this design and require a new feasibility assessment.

Ways to potentially overcome these barriers with future study design may be to undertake a more pragmatic approach. Modifiable aspects of the trial would include elements prior to drug dispensing. Case finding from primary care could be undertaken in a manner used in the CONTACT trial8 eligible patients who have had gout flares are contacted regularly and consented remotely in anticipation of a flare, streamlining as much trial activity prior to attending secondary care for assessment and dispensing of trial drug. These changes would have to considered in a subsequent feasibility assessment and would preclude progression of this design to a definitive trial.

4) Interpretation of exploratory efficacy signals

Language has been tightened in the results and discussion to emphasise hypothesis generation – a fair point.

Anakinra appeared to improve VAS score earlier than Depo-Medrone, a power calculation for a trial of superiority that may confirm this once recruitment barriers have been overcome has been suggested in the supplement.

5) Outcome measures and assessment burden

A line has been added in the methods section as the rationale of using overlapping assessments.

The rationale for the use of possible overlapping assessments was to enable reconciling assessments for a subsequent definitive study based on completion and responsiveness to treatment effect.

We have given a line in the discussion suggesting the reconciled recommendation of VAS and EQ-5D-5L.

We suggest using the EQ-5D-5L at days 1, 5 and week 8 with economic assessment using health records.

6) Safety reporting in a high-risk population

A section has been added to the conclusion about the imbalance and potential impact.

There was an imbalance in safety events between groups as one individual reported multiple adverse events. The nature of the double blind, double dummy design means that this cannot be fully dismissed, however, the non-specific nature of the symptoms and the low participant number make it difficult to interpret conclusively. We feel that this imbalance in events should not preclude a larger trial but could be mentioned in an investigator brochure for awareness.

Minor comments

These have been addressed.

Reviewer 2

Economic analysis

We have added a small paragraph on the economic analysis in the conclusion stating the context of the treatment cost differences in the context of the large secondary care costs,

Anakinra is more expensive than steroids but overall cost for health use in this group of patients is high, especially secondary care costs. It would be important for the definitive trial to include an economic evaluation so that any additional costs could be considered alongside potential savings from reductions in length of stay and any health benefits that may arise from anakinra.
