# Supplementary material for: Anakinra versus intramuscular methylprednisolone acetate for gout flares in people with chronic kidney disease (ASGARD): a UK randomised multicentre, double-blind, double-dummy controlled feasibility study with economic, qualitative and safety assessment
Source: BMJ Open. 2026 Sep 30;16(9):e114454. doi: 10.1136/bmjopen-2025-114454 (PMC13629947; doi:10.1136/bmjopen-2025-114454)
Supplement: Supplementary file 3 [file bmjopen-16-9-s002.docx]

Supplement

Contents

1. Eligibility criteria	2
2. Harms	5
3. Graph showing Kaplan-Meier curve for time to 50% reduction of 0-100 VAS from baseline.	7
   3.3 Proposed power calculation or future study	8
4. Table showing Likert scores between groups.	9
5. Table showing comparison of trends in functional assessment and

quality of life between participants who received treatments.	10

6. Table showing breakdown of SF-36 by individual components.	11
   6.1 Spidergram of SF-36 by individual components	12
7. Trends in bloods results	13
8. Qualitative analysis	15

8.1 Participant interview questions

8.2 Qualitative report

9. Pre-screening report	25
10. Table of amendments	30

**1	ELIGIBILITY CRITERIA**

Inclusion criteria

1.	Subjects capable of giving informed consent.

2.	Male or non-pregnant, non-nursing female

3.	≥ 18 years of age

4.	eGFR < 60mls/min/1.73m2 as calculated using serum creatinine and modified MDRD formula or CKD-EPI, on two occasions at least two months apart with one being in the last six months. Creatinine at time of presentation can be used unless participant has an acute kidney injury as defined by serum creatinine rise by ≥ 26µmol/L within 48 hours or ≥ 1.5-fold rise from baseline value.

5.	Diagnosis of gout arthritis as defined by the American College of Rheumatology 1977 preliminary criteria.

6.	Gout attack ≤ 7 days

7.	Baseline pain intensity greater than or equal to 50mm on the 0-100 mm VAS. In the case of multiple joints (≤ 3), the most affected joint will be assessed.

Exclusion criteria

1.	Treatment with Colchicine in the last week; participant can be recruited if treatment has been for <24 hours.

2.	Initiation of or change to dose of NSAIDS in the last week; participant can be recruited if treatment has been for <24 hours. NSAID use is not an exclusion criterion if taken at a stable dose for at least four weeks.

3.	Initiation of or change to dose of systemic steroid in the last week; participant can be recruited if treatment has been for <24 hours (excludes Depo preparations). Systemic steroid use is not an exclusion criterion if taken at an equivalent dose of 10mg Prednisolone or less for the last four weeks.

4.	Polyarticular gout, i.e. affecting four or more 4 joints

5.	Rheumatoid arthritis, evidence/suspicion of infectious/septic arthritis, or other acute inflammatory arthritis such as systemic lupus erythematosus, mixed connective tissue disease, scleroderma, polymyositis, or significant systemic involvement secondary to rheumatoid arthritis.

6.	Con-current immunosuppression/immunomodulatory treatment (Calcineurin inhibitor, anti-proliferative or biologic) therapy for other reason i.e. organ transplant.

7.	Prior history or current inflammatory joint disease other than gout (e.g. rheumatoid arthritis (RA), reactive arthritis, psoriatic arthritis, seronegative spondyloarthropathy, Lyme disease).

8.	Current active malignancy (with the exception of basal cell or squamous cell carcinoma of the skin, cervical intraepithelial neoplasia and non-metastatic/advanced prostate cancer).

9.	Any patients with contra-indication to intramuscular injection such as coagulopathy or thrombocytopenia (Platelet count<100 x 109/L (100,000/mm3)).

10.	Abnormal liver function tests: Total bilirubin>upper limit of normal, Alanine aminotransferase (ALT) or Aspartate Aminotransferase (AST) >2 times upper limit of normal.

11.	Haemoglobin < 85g/L (8.5 g/dL)

12.	White blood cell (WBC) count<1.5 x 109/L (1000/mm3), absolute neutrophil count<1.5 x 109/L (1000/mm3)

13.	Evidence of active infection requiring intra-venous (IV) anti-biotic drug administration within last four weeks. Potential participants who were empirically treated with a course of intravenous antibiotics for a presumptive diagnosis of an infection may be eligible where the treating medical care team has subsequently excluded an active infection and consequently discontinued antibiotic therapy at least four hours before randomisation. These potential participants can be discussed with the investigator where clarity is required.

14.	Evidence of uncontrolled concomitant cardiovascular, nervous system, pulmonary (including obstructive pulmonary disease), hepatic, endocrine (including uncontrolled diabetes) or gastrointestinal disease. Potential participants who have active concomitant disease can only be eligible after discussion and agreement with the treating medical team.

15.	Known positive hepatitis B virus surface antigen (HBsAg), hepatitis C (HCV) antibody or HIV.

16.	Females of child bearing potential who are not willing to use highly effective birth control methods from the time of consent to one week after treatment discontinuation. Highly effective method of contraception (hormonal or barrier method of birth control; abstinence) consist of:

•	Combined (estrogen and progestogen containing) hormonal contraception associated with inhibition of ovulation; oral, intravaginal, transdermal.

•	Progestogen-only hormonal contraception associated with inhibition of ovulation; oral, injectable, implantable.

•	Intrauterine device (IUD) or intrauterine hormone-releasing system (IUS)

•	Bilateral tubal occlusion or vasectomised partner

•	Sexual abstinence, defined as true abstinence when it is in line with the preferred and usual lifestyle of the participant. Periodic abstinence (e.g. calendar, ovulation, sympthothermal, post-ovulation) and withdrawal methods are not acceptable methods of contraception.

17.	Men who are sexually active with a female partner who are not willing to use highly effective birth control methods from the time of consent to one week after treatment discontinuation.

18.	Females of childbearing potential must have a negative pregnancy test (highly sensitive urine or serum pregnancy test after a confirmed menstrual period) within 7 days prior to treatment initiation. Subjects are considered not of child bearing potential if they are surgically sterile (i.e. they have undergone a hysterectomy, bilateral tubal ligation, or bilateral oophorectomy) or they are postmenopausal.

19.	Females must not be breastfeeding.

20.	Patients who have had treatment as part of this trial cannot have repeat treatment for another flare as part of the trial.

21.	Patients with allergies to excipients of IMPs: citric acid, anhydrous, sodium chloride, disodium edetate dehydrate, polysorbate 80, sodium hydroxide. Hypersensitivity to E. Coli derived proteins, egg proteins and soy proteins. Patients with a latex allergy are also not eligible as the inner needle cover of the pre-filled syringe contains dry natural rubber (a derivative of latex).

**2. Harms**

| Adverse event | Anakinra | Depo-Medrone |
| --- | --- | --- |
| Total | 14 | 5 |
| Nausea and vomiting |  | 1 (moderate) |
| Diarrhoea | 2* (mild) episodes reported by the same patient at day 1 and day 10. |  |
| Shortness of breath | 1*(mild) | 1 (moderate) |
| Cough | 2*(mild) |  |
| Fatigue | 1*(mild) |  |
| Thrush | 1*(mild) |  |
| Sore throat | 1(mild) |  |
| Runny nose / congestion | 2*(mild) |  |
| Dizziness |  | 1(mild) |
| Fall |  | 1(mild) |
| Itchy forehead / scalp | 1* |  |
| Neck pain | 1(mild, known degenerative changes to neck) |  |
| Back pain |  | 1 (severe, known degenerative changes to back) |
| Body pains | 1 |  |
| Injection site reaction | 1 (mild) |  |

*Reported by one participant

Gout flares recorded treatment with IMP

| Anakinra | Depo-Medrone |
| --- | --- |
| - 1 patient at day 10, received oral prednisolone for 5 days. - 1 at day 14 (mild) and again at day 35 (mild). - 1 patient at 22 days (moderate). - 1 patient at 27 days (mild), received oral prednisolone for 5 days. | - 1 patient at 11 days (moderate), received oral prednisolone for 5 days. - 1 patient at day 69 (moderate). |

**3.	Graph showing Kaplan-Meier curve for time to 50% reduction of 0-100 VAS from baseline**

**3.1 Power calculation for future trial of superiority**

The sample size calculation is based on the differences in the average VAS between the two groups.

A sample size of 58 patients (29 for each group) achieves 85% power to detect a difference in the average VAS scores (average of day 2 - day 4) of 13.66 using a two-sided Mann-Whitney U test assuming that the significance level is 0.05 and the SDs are 14.43 for group1 and 18.06 for group2.

(The sample size might not be reliable as this was estimated based on 17 patients).

**4.** **Table showing Likert scores between groups**

|  | | Score | Day 1 | Day 2 | Day 3 | Day 4 | Day 5 | Day 6 | Day 7 |
| --- | --- | --- | --- | --- | --- | --- | --- | --- | --- |
| Patient assessment of pain:  1 - none, 2 - mild pain, 3 - moderate pain, 4 - severe pain, 5 - extreme pain | Anakinra  (n=8) | 1  2  3  4  5 | -  -  3  -  5 | -  2  4  -  2 | -  3  4  -  1 | -  2  6  -  - | -  4  4  -  - | 1  5  2  -  - | 2  5  1  -  - |
|  | Depo-  Medrone  (n=9) | 1  2  3  4  5 | -  -  2  -  7 | -  -  5  1  3 | 1  -  6  -  2 | 2  1  5  -  1 | 1  1  6  -  1 | 1  3  4  -  1 | 1  6  2  -  - |
| Physician assessment of joint swelling:  1 – no swelling, 2 - palpable, 3 - visible, 4 - bulging beyond margins | Anakinra  (n=8) | 1  2  3  4 | -  -  3  5 | -  2  6  - |  | | | | 4  3  1 |
|  | Depo-  Medrone  (n=9) | 1  2  3  4 | -  -  2  7 | 1  2  5  1 |  | | | | 4  5  -  - |
| Physician assessment of joint tenderness:  1 - no pain, 2 - patient states there is pain, 3 -patient states there is pain and winces, 4 - patient states there is pain. winces and withdraws) | Anakinra  (n=8) | 1  2  3  4 | -  -  3  5 | -  1  6  1 |  | | | | 7  1  -  - |
|  | Depo-  Medrone  (n=9) | 1  2  3  4 | -  -  4  5 | 1  1  3  4 |  | | | | 3  2  4  - |

5. **Table showing comparison of trends in functional assessment and quality of life between participants who received treatments.**

|  | VAS | | LEFS | | HAQ-DI | | EQ-5D-5L | | SF36 (PCS) | | SF 36 (MCS) | |
| --- | --- | --- | --- | --- | --- | --- | --- | --- | --- | --- | --- | --- |
|  | Anakinra  (n=8) | Depo-  Medrone  (n=9) | Anakinra  (n=8) | Depo-  Medrone  (n=9) | Anakinra  (n=8) | Depo-  Medrone  (n=9) | Anakinra  (n=8) | Depo-  Medrone  (n=9) | Anakinra  (n=8) | Depo-  Medrone  (n=9) | Anakinra  (n=8) | Depo-  Medrone  (n=9) |
| Day1 | 70  (61-74) | 70  (61-73) | 27.5  (22-39) | 19.5  (7-30.5) | 1.3  (0.5-1.4) | 1.3  (0.9-2) | 0.4  (0.1-0.7) | 0.1  (-0.002-0.5) | 22.9  (21.8-35.1) | 22.7  (19.1-26.2) | 42.2  (38-54.2) | 46.3  (37.7-59.5) |
| Day2 | 43  (30-55.5) | 59  (56-70) | 30  (20.5-  39.5) | 16  (7-29) |  | | 0.6  (0.2-0.7) | 0.5  (0.2-0.6) |  | | | |
| Day7 | 23.5  (12.5-28) | 23  (11-27) | 34  (32-44) | 23  (19-57) | 0.9  (0.8-1.3) | 1.1  (0-1.5) | 0.6  (0.58-0.7) | 0.7  (0.6-0.74) | 29.2  (26.5-32) | 29  (22.8-31.3) | 39.8  (30.1-51.5) | 51.7  (36.4-56.7) |
| Week 8 |  | | | | 1.2  (0.6-1.6) | 1.3  (0.3-2.5) | 0.6  (0.3-0.8) | 0.7  (0.2-0.72) | 32.3  (28.8-40.3) | 30.7  (21.2-45.7) | 43.6  (25.2-50.3) | 53  (44.7-56.8) |

6. **Table showing breakdown of SF-36 by individual components Mean (SD)**

| Outcomes | Day 1 | | Day 7 | | Week 8 | |
| --- | --- | --- | --- | --- | --- | --- |
|  | Anakinra  (n=8) | Depo-  Medrone  (n=9) | Anakinra  (n=8) | Depo-  Medrone  (n=9) | Anakinra  (n=8) | Depo-  Medrone  (n=9) |
| Physical functioning | 28.8 (14.3) | 26.7 (26.5) | 32.5 (10) | 35 (32.2) | 33.8 (24.3) | 32.2 (33.6) |
| Role limitations due to physical health | 21.9 (31.2) | 12.5 (35.4) | 18.8 (34.7) | 27.8 (44.1) | 43.8 (47.7) | 50 (50) |
| Role limitations due to emotional problems | 50 (43.6) | 50 (53.5) | 37.5 (45.2) | 51.9 (50.3) | 29.2 (41.5) | 66.7 (50) |
| Energy/fatigue | 41.9 (20.7) | 35.6 (18.8) | 38.1 (25.8) | 35 (20.8) | 36.9 (26.0) | 34.4 (27.4) |
| Emotional well-being | 61 (13.6) | 62.2 (22.3) | 55 (25.2) | 66.7 (18.4) | 59.5 (25.3) | 67.6 (19.6) |
| Social functioning | 40.6 (29.7) | 54.2 (39.0) | 42.2 (28.3) | 65.3 (29.8) | 48.4 (29.5) | 68.8 (26.7) |
| Pain | 35.6 (20.9) | 33.1 (24.5) | 39.1 (23.2) | 40.3 (28.8) | 54.1 (35.6) | 49.2 (35.3) |
| General health | 39.4 (19.7) | 40 (18.0) | 46.9 (14.1) | 49.4 (15.7) | 31.9 (20.2) | 37.8 (17.5) |

6.1 **Figure showing spidergram of SF-36 scores comparing patients treated with Anakinra (left) and Depo-Medrone (right)**

**7.Trends in blood results**

Graph showing serum creatinine trend (mean ± SD: day1, day2, day 7 and week 8) between Anakinra (Group 1) and Depo-Medrone (Group 2)

Graph showing serum CRP trend (mean ± SD: day1, day2, day 7 and week 8) between Anakinra (Group 1) and Depo-Medrone (Group 2)

Graph showing blood white cell counts (x 10^9^) trend (mean ± SD: day1, day2, day 7 and week 8) between Anakinra (Group 1) and Depo-Medrone (Group 2)

Graph showing neutrophil counts (x 10^9^) trend (mean ± SD: day1, day2, day 7 and week 8) between Anakinra (Group 1) and Depo-Medrone (Group 2)

8. **ASGARD - Qualitative Analysis**

**Participant Interview Guide**

**1. Demographic Information**

How old are you?

What is your gender?

**2. Domestic Situation**

Could you tell me a little about your current living situation?

Do you live alone, with a partner, family members, or others?

**3. Referral to Asgard**

How did you first hear about the Asgard trial?

Who referred you to the study?

What were your reasons for deciding to take part?

**4. Recruitment Process and Informed Consent**

Can you describe your experience of joining the study?

How clear was the information provided to you before you agreed to participate?

Did you feel you had enough opportunity to ask questions before consenting?

What was your understanding of the purpose of the trial?

What was your understanding of randomisation within the trial?

Were there any aspects of the recruitment process that were confusing or unclear?

**5. Experience of Self-Injection and Diary Completion**

Before joining the trial, had you ever self-injected medication?

How did you find the training or instructions provided for self-injection?

Can you describe your experience of self-injecting during the trial?

Were there any challenges or concerns related to self-injection?

How confident did you feel carrying out the injections?

How did you find keeping the study diary?

Were the diary requirements easy or difficult to complete?

Did anything make it harder to maintain the diary consistently?

**6. Experience of Participating in the Trial**

Overall, how would you describe your experience of taking part in the trial?

What aspects of the trial worked well from your perspective?

Were there any aspects you found difficult, inconvenient, or burdensome?

How supported did you feel by the study team throughout your participation?

Did participation have any impact on your daily life?

**7. Perception of Trial Allocation**

During the study, did you think you knew which trial arm you had been allocated to?

What made you think that?

How confident were you in your belief?

Did your perception of allocation affect your experience of the trial in any way?

**8. Suggestions for Improvement**

What, if anything, could have been done differently to improve your experience of the trial?

Are there any changes you would recommend to the recruitment process?

Are there any changes you would recommend to the self-injection training or support provided?

How could the diary process be improved?

What advice would you give to researchers running a similar trial in the future?

**Closing Question**

**Is there anything else about your experience of participating in the Asgard trial that you would like to share that we have not discussed?**

Thank participant for their time and contribution.

**Qualitative report**

**Introduction**

Seven study participants were interviewed by telephone to gain a qualitative understanding of their experience of participating in the trial. This was reduced from a planned 12 interviewees due to the early curtailment of recruitment into the study. Four women and three men living in various types of domestic situation (with/without spouse/car) were interviewed. Ages ranged from 56 to 81. One interview (no. 3) was conducted with the patient’s husband as the patient was herself too tired to undertake a telephone call. Several key findings are relevant to any future large scale study design but generally interviewees reported that they were satisfied with how the trial worked for them and did not identify fundamental issues that they thought undermined the feasibility of the trial.

**Key findings**

Overall, interviewees were all very positive about their experience of taking part in the trial. Almost universally they said that the patient information they were given was clear and comprehensive. All emphasised the care and time that the recruiting clinicians put into answering their questions.

Participants reported the time from recruitment to receiving their first injection on day one was lengthy, up to 6 or 7 hours. Consideration should be given on how to reduce this in any future full-scale trial. Patients generally self-injected at home with no problem and felt their preparation for this by clinicians gave them the confidence needed to do so without worry. Around half of patients found keeping the daily diary burdensome and frustrating to the point where they said they did not consistently complete it. Consideration should therefore be given in any future trial to reducing the frequency of diary completion, reducing the number of questions asked and emphasising its importance more explicitly by recruiting clinicians.

**The interviewees**

| **Interview no.** | **Gender** | **Age range** | **Previous history of Gout** | **Domestic circumstances – spouse/car** | **Recruitment pathway** | **Treat-ment arm** | **Perception of treatment received** |
| --- | --- | --- | --- | --- | --- | --- | --- |
| 1 | Female | 50-59 | Yes | Not known | GP | 2 | None |
| 2 | Female | 70-79 | No | Not known | A&E | 2 | None |
| 3 | Female* | 80-89 | Yes | Living with spouse, car | GP | 2 | None |
| 4 | Male | 70-79 | Yes | Car | GP | 2 | None |
| 5 | Male | 70-79 | Yes | Living with spouse, car | Hospital Specialist | 1 | None |
| 6 | Male | 70-79 | Yes | Living with spouse, car | Hospital Specialist | 2 | None |
| 7 | Female | 70-79 | No | Lives alone, no car | A&E | 2 | None |

*****Interview conducted with patient’s husband due to patient’s tiredness

**Recruitment pathway**

Interviewees were recruited into the trial through several pathways. Three patients who had a history of gout attacks and flare ups were told about the trial by their GP. Two patients who had not had previous gout attacks were recruited into the trial after visiting A&E departments with their first gout attacks. Two patients with a pre-existing history of gout were recruited through their hospital consultant or specialist.

**Patient information and informed consent process**

The patient information materials and informed consent process appears to be robust and was welcomed by patients.

Six out of the seven patients interviewed said that they thought the patient information they received, both written and verbal, was clear, comprehensive and pitched at the right level.

“The information was very helpful. They explained it to me very well … they explained everything at the hospital. The nurse there was X and she dealt with me very personally so it felt like any problems I could go back to her. So she sat down and took the time to explain, it wasn’t a rushed thing… ” (Interview no 1)

“I was able to clearly understand it because before that I saw the leaflet and was thinking about doing it and had read all the literature. But really, at the beginning it was a bit worrying because I thought Oh injections and hospitals, and I had a holiday booked, but when I did get there and met Nurse E she explained it so much better because like she said I had to do it for seven days but I only had to come in the first day, some of it would be the real thing but I wouldn’t know if it was the real thing and all the rest of it.” (Interview no 1)

“It was all explained really clearly. Made very clear (Interview no 5)

“Dr X explained that he would not know which treatment she would be given. As a layman he explained it to down to our level … he used our language. It took quite a long time, maybe an hour and a half to explain it …” (Interview 3 - proxy interviewee)

“The GP didn’t explain anything in detail. But Dr X and the nurse I think explained it very, very well. Dr G explained it very well and put us very much at ease. (interview no 2)

Probing questions revealed that generally interviewees understood the purpose of the trial and key aspects of the trial design, including what “randomisation” and “double blind” meant in practice.

“They explained very clearly that no one would know what I was getting, not even the doctors. And that it was random.” (interview no 5)

Just one exception was an interviewee who did not appear to fully understand what she had been told about randomisation, although she did say she was told about it. She also said that the double-blind nature of the trial had not been explained to her.

“They told me about the randomisation, but I didn’t really get it. I don’t remember them saying anything about double blind. I just went along with anything they wanted me to do as I was in so much pain” (interview no 7)

This interviewee went on to say that she had no recollection of the process of giving informed consent, although she accepted that they must have done so in order to be in the trial.

**Trial reality vs trial expectations – the value of direct contact with clinicians**

Interviewees generally felt that the reality of taking part in the trial matched their expectations, especially after they had the chance to talk directly to the doctors and nurses taking informed consent. All interviewees mentioned how much care and time was taken to explain the trial to them and how reassuring this personal attention was.

“When I was looking at the trial, when I first read the literature, it seemed a little bit scary because it was like you’re going to be taking part in a hospital thing, you have to be in a hospital every day, you have to inject yourself every day and you won’t know whether its real or not and it just sounded like you’re in an experiment so it became a bit of a worry. But when I did get to the hospital and I met the nurse and doctor it didn’t seem so scary and it seemed like it was better to take part and hopefully help other patients, so then I was really pleased to be participating” (interview no 1)

“It was all very much how it was explained to me. The explanations were really very good. I was never in any doubt at all. I was very pleased to be part of it and its helped my gout a lot because I see Dr X now and he has put me on Allopurinol and touch wood I haven’t had an attack since” (interview no 4)

“Nurse E made it so clear. I couldn’t fault her one bit. A nurse like that that really does explain everything so well. She was really very good (interview no 5)

“There were no nasty surprises” (interview no 7)

**Day 1: A long wait from recruitment to first injection**

Study patients reported considerable time frames on day 1 from recruitment into the study, through to their first injection with several reporting long waits for urine samples and assessments to be taken. Some found this frustrating but at the same time there was a general forbearance and understanding of the pressures on NHS staffing.

“Day one was a bit of a long wait because I was waiting for ages to get medicine because what happened was the doctor was a bit busy, and the pharmacist in the hospital was not here. And that’s what took the time, getting the medication. So, I got the medication, I was told how to inject myself and I got the first injection. So that was basically … (Baby interrupts) …. I was there from about 1 to about 6 o’clock. (Interview no 1).

“It was quite a long process of filling in all the forms. 2 or 3 hours. I think the first day went very well, it was some of the follow-on visits were not so good” (interview no 4)

“I was there for 5 hours. That’s a long time, isn’t it?” (Interview no 5)

For one interviewee the long wait for medication provided an opportunity for her to gain more understanding about the trial from the research nurse:

“She must have spent a good two or three hours...because I was there for so long because I was also waiting for the medication, so she was doing what she was doing but she would still come back to me, she would sit and explain...

For this interviewee however, the long wait for medication nearly caused her to withdraw from the trial because of the pain and discomfort she was in. Ultimately, it was the care and encouragement of the research nurse that ensured she did not drop out.

“The only thing that I think could have been better would on the first day not to have spent five hours hanging around. I was in a lot of pain. I think what should have been done is they would have the medication more ready for access because that was the problem. We was waiting for the medication for like three hours. That was the hold up. Because the doctor signed for it, then they had to send it to the pharmacy, and then they had to get the medication and send it back to me. So for that time I was waiting...

I think that nearly put me off while I was sitting there. The pain was getting worse and I had driven there and I didn’t know if I was going to be able to drive home ... I nearly gave up on it. But because of the nurse I didn’t. She just kept saying just wait, and she put me in a comfy chair and she come through with a blanket .. that’s why I hanged out. If it wasn’t for that I would have gone home.” (interview no 1)

Considerations could be made as to how to smooth and speed the pathway from recruitment into the study through to receiving the first injection. Considering the multiple tasks that need to be accomplished on day 1 it may also be worthwhile to make very clear to potential participants the realistic timeframe from recruitment through to being able to leave the hospital on day 1 in order that they can be mentally prepared for a long wait. The personal care and attention of research staff was a key mitigating factor for patients enduring long waits for their medication.

**Self-injection**

The trial demonstrated that it is feasible to ask patients to self-inject at home. While the first- and second-day’s injections were done at the hospital, patients were typically required to start self-injecting at home from day 3. Patients reported that this was accomplished without any problems. Several emphasized the confidence that clinicians who showed them how to inject themselves, gave them.

“I had never given a self-injection. That was a bit worrying. But after I was shown how to do it, it never became a problem. The hospital did the first injection. I felt the nurse that was there was brilliant. Because she was able to explain everything that I needed to know. I was a bit concerned about the injection but she said, look I’ll show you how to do it, and then the next day I had to go in she told me to bring one of the injections with me, and then I had to do it. (Interview no 1)

“I was very confident. It was fine. It wasn’t as painful as I thought. It wasn’t like getting a blood test or it was quite a small needle and I didn’t really feel much of it. It was fine.” (Interview no 1)

“I was very confident on the self-injections. I had injected my wife before and it was very easy because of that.” (interview no 5)

**Completing the diary**

Participants were asked to keep a pain diary from day 3. About half of interviewees reported that this was completed consistently and without problem. Several linked it to doing their injections.

“I had no problems giving injection on any day. I remembered to do the diary by doing the diary straight after the injections”. (interview no 1)

I successfully injected on every day and successfully completed the diary on every single day (interview no 5).

One participant said that they returned to hospital between each injection where questionnaires were completed and that they did not keep a diary.

Other interviewees found keeping the diary burdensome and several were frustrated that they were asked the same questions on each day of the diary. Several felt this was a waste of time and did not appear to fully understand why they were being asked to do it.

“I couldn’t be bothered to do it myself. The nurses helped me to do it. It was the same questions every day … silly questions” (interview no 7 – hospital inpatient for first days of trial)

“I found the questionnaires on day 2 were more or less the same as day one. I didn’t really understand why we were doing the same again (interview no 3 - proxy).

For any future trial there may be value in both reducing the patient burden in terms of the number of days that a diary needs to be completed and reducing the number of questions. Clearer patient information at the point of recruitment may be needed and building this into verbal explanation by the consenting clinician may be beneficial to adherence.

**Perception of treatment received**

Most interviewees had no idea what treatment they thought they had received. When asked if they had any idea typical responses were:

“We have no idea what she had. She recovered about the same as when treated with steroids. She’s had a couple of bouts since and received steroids that have cleared it up quickly (interview no 3 - proxy)

“No idea. It could have been water for all I know” (interview no 4)

“None. but I would be interested to know” (interview no 5)

Just one interviewee thought that she might know which treatment she had received,

“The gout stayed a little longer, erm and in fact after the seven days I was just recovering, usually with medication it takes about two days to improve but this time here it took a few more days for the pain to start to subside. I was also told to take the medication for the gout if it became really painful. But I don’t really know. Usually when I go to the GP and get tablets it works quite quickly so maybe I got the fake one?” (interview no 1)

**Is there anything that would make participation better for future patients?**

Only one patient volunteered a potential improvement to the study design and this focused on where she was sent for blood tests.

“They kept asking for blood tests. Which is a transport issue for me. They kept sending me to different places. Why couldn’t I have my blood tests done local to me at my local blood test centre. It all adds to stress.” (interview no 7)

Generally, however, patients were very positive about the experience of taking part in the trial and most did not have anything they felt could be improved other than waiting times on the first day and a reduction in the diary burden.

“I honestly don’t think so. I am being very straight with you. It was a very happy experience. It was quite a long day but it was a happy experience because everybody was very helpful and very kind, and very clear in explaining what was going on so there really were not any issues at all.” (interview no 4)

“No, not that I can think of. It was all really good. Excellent in fact” (interview no 6)

“I think it was as good as it could be” (interview no 3 – proxy respondent)

“It would be nice to get the feedback to see which treatment I got” (interview no 1)

**Summary**

Generally, interviewees were very positive about the study and their own participation. Three key considerations to be considered in any future trial are:

1. Streamlining hospital procedures on day 1 to reduce what was a very long and extended day for some patients. The length of time taken for patients to get to medication can also be mitigated with more explicit information on how long the full process might take and research staff support and care.

2. Development of a more streamlined diary to reduce the patient burden combined with clearer information on the importance of its full completion for the integrity of the study to the study.
3. Feedback to participants post-trial as to which treatment they actually received would be welcomed.
9. **Screening data analysis**

**ASGARD study: Analysis of screening data**

**Description of pre-screening and screening data**

Seven patients were formally screened with consent during the initial start, and it was found that 3 were not eligible due to eGFR not being low enough, two unlikely to have gout, one was on colchicine, and one was in heart failure. It was felt that a pre-screening / screening amendment without consent would aid the study design and allow for database searched in primary care / community. In March 2017, there was an amendment to the protocol which introduced a pre-screening process for basic eligibility prior to consent. Pre-screening is defined in the protocol (V1.9 13Apr21) as potential participants undergoing: ‘*an initial pre-screening process for basic eligibility; inclusion and exclusion. This will include a review of renal function and information on previous testing for gout diagnosis such as uric acid, and findings of imaging and joint fluid analysis. This will help avoid ineligible participants undergoing a baseline assessment and consent process who can be discounted as being ineligible early on.’* This pre-screening process proceeded a baseline assessment for eligibility.

**Recorded screening information across hospital sites**

Available screening data at each of the hospital sites are summarised in **Table 1** below.

Guys and St Thomas Hospital (GSTT) had separate pre-screening and screening data logs, West Suffolk hospital (WSH) kept a pre-screening log only, and data available for Kings College Hospital (KCH) and Southend Hospital (SUH) were not differentiated between screening and pre-screening activities (all screening data for SUH are taken from the MACRO database).

**Table 1:** Number of patients screened/pre-screened at each hospital site

|  | Pre-screened |  | Screened | Pre-S/Screened | **Total** |
| --- | --- | --- | --- | --- | --- |
| GSTT | 46 |  | 53 |  | **99** |
| KCH |  |  |  | 38 | **38** |
| SUH |  |  |  | 137 | **137** |
| WSH | 36 |  |  |  | **36** |
| **Total** | **82** |  | **53** | **175** | **310** |

**Recruitment and eligibility**

Of the 310 patients included in the screening logs, 21 were eligible to take part and recruited. Of the remaining 289 the data provided either a single criterion for non-eligibility (*n* = 186), or multiple criteria (*n* = 17), or no criteria (included in this group are patients where data were not available on reasons for non-eligibility, and patients approached and/or given a PIS who either did not respond, or declined to participate, or could not be contacted). These data are summarised in **Figures 1, 2 and 3** below.

**Summary of findings**

For those patients for who there were reasons available for their non-eligibility; the most common reasons were: not CKD (101/203 – **49.8%**), no gout diagnosis (19/203 – **9.4%**), no gout flare (17/203 – **8.4%**), Colchicine (17/203 – **8.4%**), concomitant disease (10/203 – **4.9%**), arthritis (9/203 – **4.4%**), steroids (8/203 – **3.9%**), active malignancy (8/203 – **3.9%**).

****NOTE:** No screening data are on file for Lister Hospital or Princess Alexandra Hospital.

Number of screened participants

| Reason for non-eligibility | Total |
| --- | --- |
| Not CKD, eGFR outside limits (I4) | 96 |
| No gout flare (I6) | 26 |
| No gout diagnosis (I5) | 14 |
| Colchicine (E1) | 11 |
| Concomitant disease (E14) | 9 |
| Arthritis (E5) | 7 |
| Steroids (E3) | 7 |
| Immuno treatment / transplant (E6) | 5 |
| Active malignancy (E8) | 4 |
| Hepatitis B, HIV (E15) | 2 |
| Infection /IV antibiotics (E13) | 2 |
| Other inflamm joint disease (E7) | 1 |
| Low Hb (E11) | 1 |
| Polyarticular gout (E4) | 1 |

**Figure 1:** Number of patients deemed ineligible based on a single inclusion/exclusion criterion

Number of screened participants

| Reason for non-eligibility | Total |
| --- | --- |
| No gout; colchicine (I5; E1) | 2 |
| Pain <50mm; inf / IV abx (I7, E13) | 2 |
| No gout; active malignancy (I5, E8) | 2 |
| Not CKD; no gout flare (I4, I6) | 1 |
| Not CKD; arthritis (I4, E5) | 1 |
| Not CKD; Hep B (I4, E15) | 1 |
| Arthritis; active malignancy (E5, E8) | 1 |
| Not CKD; no gout (I4, I5) | 1 |
| Colchicine; prednisolone (E1, E3) | 1 |
| Polyarticular gout; colchicine (E4, E1) | 1 |
| Concomitant disease; abn LFT (E14, E10) | 1 |
| Active malign; low Hb; abn LFTs (E8, E11, E10 ) | 1 |
| Colchicine; NSAIDs (E1, E2) | 1 |
| Not CKD; colchicine (I4, E1) | 1 |

**Figure 2:** Number of patients deemed ineligible based on a multiple inclusion/exclusion criteria

Number of screened patients

| Reason for non-eligibilty | Total |
| --- | --- |
| Reason No information available | 30 |
| Reason PIS sent - no response/info available | 28 |
| Reason Patient declined | 17 |
| Reason Patient not able to take part | 5 |
| Reason patient contacted - message left | 2 |
| Reason Patient could not be contacted | 4 |

**Figure 3:** Number of patients where data were not available on reasons for non-eligibility

10. **Table of amendments**

| **Amendment No.** | **Protocol version no.** | **Date issued** | **Author(s) of changes** | **Details of changes made** |
| --- | --- | --- | --- | --- |
| 1 | 1.1 | 17/11/15 | G Balasubramaniam | Removal of day 4 assessment due to feedback from recruiting centres and outcome of TMG discussion. Week 8 visit for all patients to record health resource use and blood tests. |
| 2 | 1.2 | 01/01/16 | G Balasubramaniam | Exclusion criteria amended to be clear to exclude patients requiring intra-venous anti-biotic administration, or within two weeks of such treatment.  Abstinence from sex clarified as true abstinence i.e. non-periodic (calendar, ovulation, sympthothermal, post-ovulation methods)  Included a rationale for dose and dose duration for Anakinra (Kineret) |
| 3 | 1.3 | 13/05/16 | G Balasubramaniam | Trial pharmacist changed from Stuart Chandler to trial pharmacists: Amiirah Ropun, Inisa Idress and Laura Wilson.  Trial statistician changed from Michael Parker to Jufen Zhang. |
| 4 | 1.3 | 01/07/16 | G Balasubramaniam | Information in baseline assessment pertaining to new diagnostic gout criteria in 2015 (ACR and EULAR) taken in baseline assessment. |
| 5 | 1.3 | 01/07/16 | G Balasubramaniam | Use of CKD EPI or MDRD (as per local guidelines) to calculate eGFR |
| 6 | 1.3 | 01/07/16 | G Balasubramaniam | Men of child bearing age not willing to undertake highly effective contraception during treatment period excluded |
| 7 | 1.3 | 01/07/16 | G Balasubramaniam | One further centre added Basildon and Thurrock University Hospital NHS Trust. |
| 8 | 1.3 | 01/07/16 | G Balasubramaniam | PI changed at Mid Essex NHS Trust |
| 9 | 1.3 | 01/07/16 | G Balasubramaniam | Participants can be contacted on Friday and Monday as a reminder for weekend or compliance check respectively. |
| 10 | 1.3 | 01/07/16 | G Balasubramaniam | Baseline assessments can be made after four hours as opposed to six hours if permitted form of analgesia used. |
| 11 | 1.3 | 01/07/16 | G Balasubramaniam | ARCTU email added in SAE / SUSAR section to help with monitoring. |
| 12 | 1.4 | 01/02/17 | G Balasubramaniam | Depo-Medrone changed to generic term of intramuscular methylprednisolone throughout protocol. |
| 13 | 1.4 | 01/02/17 | G Balasubramaniam | Exclusion criteria changed to specific criteria for each treatment i.e. Colchicine, Steroids and NSAIDS |
| 14 | 1.4 | 01/02/17 | G Balasubramaniam | Line in exclusion criteria 5, (7 in new version 1.4) “Con-current medication for RA like methotrexate and anti-TNF treatment has been associated with increased risk of neutropenia and infection” removed as repeats exclusion criteria 4 (6 in version 1.4) |
| 15 | 1.4 | 01/02/17 | G Balasubramaniam | Exclusion criteria 13; potential participants who were given empirical treatment with antibiotics but infection has been excluded can be recruited. |
| 16 | 1.4 | 01/02/17 | G Balasubramaniam | Exclusion criteria 14; potential participants uncontrolled concomitant disease. The term “serious” has been removed and potential participation in the trial should be in agreement with the direct medical team. |
| 17 | 1.4 | 01/02/17 | G Balasubramaniam | Pre-screening for potential participants. |
| 18 | 1.4 | 01/02/17 | G Balasubramaniam | Use of present renal blood tests that may show an acute kidney injury will not be used to calculate baseline renal function. |
| 19 | 1.5 | 13/03/2017 | G Balasubramaniam | Trial Manager changed from Trisha Parker to Arit Udoh. |
| 20 | 1.5 | 13/03/2017 | G Balasubramaniam | Section 7.13 Rescue medication added. Rescue treatment is Prednisolone 35mg po orally, with additional low dose colchicine if needed. |
| 21 | 1.5 | 13/03/2017 | G Balasubramaniam | Typographical error with blood collection in page 41. |
| 22 | 1.5 | 01/07/2017 | G Balasubramaniam | Inclusion criteria change, attack can be up to 7 days. |
| 23 | 1.5 | 01/07/2017 | G Balasubramaniam | Exclusion criteria change, participants can be included if treatment with colchicine, NSAIDS or steroids (Not depo) has been less than 24 hours. |
| 24 | 1.5 | 01/07/2017 | G Balasubramaniam | New version of Health Resource use questionnaire. |
| 25 | 1.6 | 01/08/2018 | G Balasubramaniam | Inclusion criteria to include eGFR < 30 with alternate day dosing.  **Trial Summary (page 9)**  Trial Participants  Patients with Acute Gout Attacks and Kidney Disease (eGFR < 60)  Administration Schedule  Group A (Experimental) Anakinra 100mg s/c injection D1 to D5 (alternate days for eGFR < 30) and placebo i/m methylprednisolone (Lipofundin) injection at D1  Group B (Active comparator) Intramuscular methylprednisolone (Depo-Medrone) 120mg i/m injection at D1 and placebo Anakinra s/c D1 to D5 (alternate days for eGFR < 30).  Formulation, Dose, Route of Administration  Anakinra: Pre-filled syringes 0.67mLs, 100mg s/c for 5 days. Alternate days for eGFR < 30 i.e. D1, D3, D5.  **Trial flowchart (page 19)**  Group A: Anakinra group. N=16 Anakinra 100mg s/c D1 to D5 (Alternate days if eGFR < 30, i.e. D1, D3, D5)  Placebo i/m D1  Group B: Methylprednisolone group. N=16 Methylprednisolone (Depo-Medrone) 120mg i/m D1 Placebo s/c D1 to D5 (Alternate days if eGFR < 30)  **Schedule of procedures (page 20)**  Drug administration (D1, D3, D5 if eGFR < 30)  **Study Protocol (page 22)**  A study to determine the feasibility of undertaking a definitive randomised multi-centre, double-blind, double-dummy controlled study of a novel agent Anakinra, an IL-1 receptor antagonist vs. intramuscular methylprednisolone (Depo-Medrone) for acute gout attacks in patients with ~~moderate~~ chronic kidney disease.  **1 Background: The IMPs: Anakinra (page 23)**  We want to explore the use of Anakinra for acute gout in patients with moderate renal impairment in comparison to the current standard therapy of steroids. We feel that IL-1RA inhibition provides a very specific treatment option with less side-effect. This is a timely study that aims to establish a firm evidence base for the management of a relatively common condition in a high risk group that is associated with considerable morbidity and costs.  There has been an update on the summary of product characteristics in June 2018 by the manufacturer where in patients with severe renal impairment (eGFR < 30) or end stage renal disease, including dialysis, administration of prescribed dose of Anakinra every other day should be considered.  **Rationale**  2.1 Assessment and management of risk (page 26)  ~~We have intentionally avoided using Anakinra in patients with severe renal failure (eGFR < 30mls/min/1.73m~~^~~2~~^~~) due to concerns about accumulation in pharmacokinetic studies. We are taking caution even though it has been used safely in these patients and we have some experience in our centre.~~ We are including potential participants with severe kidney disease (i.e. eGFR < 30) as pharmacokinetic evidence suggests that alternate day dosing should be suitable, and it has been suggested by the manufacturer. Increasing reports of dosing in patients with severe kidney disease show good tolerability with few adverse events. It has also been described in patients on haemodialysis using an alternate day regime where no adverse complications were noted.  **2.2 Rationale for dose and dose regimen (page 26)**  The rationale for the use of 100mg s/c for 5 days comes from emerging data where Anakinra has been used to treat acute gout. Some reports vary between 3 to 5 days but most use 5 days’ duration of treatment to ensure adequate response in all patients17-23. This includes our own experience23 where we have used 5 days’ duration of treatment to have a standard approach to all patients, exclude patients with severe kidney disease (eGFR <30 – an alternate day injection regime is used). We now have amendment from the manufacturers on the last update of the summary of product characteristics (SmPC) stating that patients with renal impairment of eGFR < 30, alternate day dosing can be considered. This was updated in June 2018.  **3.1 Primary objective (page 27)**  The aim of this study is to determine the feasibility of running a phase III double-blind, double-dummy randomised controlled trial comparing intramuscular methylprednisolone (Depo-Medrone) 120mg vs. Anakinra 100mg subcutaneous injection for 5 days for the treatment of acute gout attacks in patients with chronic kidney disease as defined by an eGFR < 60mls/min/1.73m^2^, ~~and ≥ 30mls/min/1.73m~~^~~2~~^~~.~~ with alternate day dosing for eGFR < 30mls/min/1.73m^2^ (i.e. D1, D3, D5).  **6 Eligibility criteria: 6.1 Inclusion criteria (page 31)**  4. eGFR < 60 mls/min/1.73m2 ~~and > 30 mls/min~~  **7.3.1 Method of implementing the allocation sequence (page 36)**  Participants will be randomised to one of two treatment arms, Group A and Group B:  • Group A: Anakinra 100mg s/c Day 1 to Day 5 (alternate days if eGFR < 30mls/min/1.73m^2^) and placebo intramuscular methylprednisolone (Lipofundin) 120mg i/m Day 1.  • Group B: Intramuscular methylprednisolone (Depo-Medrone) 120mg i/m Day 1 and placebo Anakinra 100mg s/c Da 1 to Day 5 (alternate days if eGFR < 30mls/min/1.73m^2^).  **8.1.3 Summary of Product Characteristics (SmPC) (page 45)**  ~~Available from www.kineretx.com/fileadmin/user/pdfs/Kineret_Full_Prescribing_Information.pdf Issue date: 12/2012~~  Available from http://www.ema.europa.eu/docs/en_GB/document_library/EPAR_-_Product_Information/human/003819/WC500187504.pdf  Update 25/06/2018  **8.1.6 Dosage schedules (page 47)**  At recruitment and randomisation, participants enrolled into the Anakinra arm will have a D1 SC injection of 100mg Anakinra. This will continue daily for 5 days in total (i.e. D1 to D5). Patients in the steroid arm for investigation product will have similar regime with the equivalent placebo.  Patients with eGFR < 30mls/min/m2 will have an alternate day regime with a dose administered on day 1, day 3 and day 5. |
| 26 | 1.6 | 01/08/2018 | G Balasubramaniam | Trial pharmacist only stated as Amirrah Ropun |
| 27 | 1.6 | 01/08/2018 | G Balasubramaniam | Guys and St Thomas’ added with Dr Ng as PI.  Kings College Hospital added as site with Dr James Galloway as PI |
| 28 | 1.7 | 01/12/2018 | G Balasubramaninm | No-cost extension, study end date 01/10/2019 |
